# Context-dependency of arbuscular mycorrhizal fungi on plant-insect interactions in an agroecosystem

**DOI:** 10.3389/fpls.2013.00338

**Published:** 2013-09-05

**Authors:** Nicholas A. Barber, E. Toby Kiers, Ruth V. Hazzard, Lynn S. Adler

**Affiliations:** ^1^Department of Biological Sciences, Northern Illinois UniversityDeKalb, IL, USA; ^2^Institute for the Study of the Environment, Sustainability, and Energy, Northern Illinois UniversityDeKalb, IL, USA; ^3^Department of Plant, Soil, and Insect Sciences, University of Massachusetts–AmherstAmherst, MA, USA; ^4^Institute of Ecological Science, Faculty of Earth and Life Sciences, Vrije Universiteit AmsterdamAmsterdam, Netherlands; ^5^Department of Biology, University of Massachusetts–AmherstAmherst, MA, USA

**Keywords:** aboveground–belowground, arbuscular mycorrhizal fungi, *Cucumis sativus*, herbivore, indirect interaction, pollinator

## Abstract

Plants interact with a variety of other community members that have the potential to indirectly influence each other through a shared host plant. Arbuscular mycorrhizal fungi (AMF) are generally considered plant mutualists because of their generally positive effects on plant nutrient status and growth. AMF may also have important indirect effects on plants by altering interactions with other community members. By influencing plant traits, AMF can modify aboveground interactions with both mutualists, such as pollinators, and antagonists, such as herbivores. Because herbivory and pollination can dramatically influence plant fitness, comprehensive assessment of plant–AMF interactions should include these indirect effects. To determine how AMF affect plant–insect interactions, we grew *Cucumis sativus* (Cucurbitaceae) under five AMF inoculum treatments and control. We measured plant growth, floral production, flower size, and foliar nutrient content of half the plants, and transferred the other half to a field setting to measure pollinator and herbivore preference of wild insects. Mycorrhizal treatment had no effect on plant biomass or floral traits but significantly affected leaf nutrients, pollinator behavior, and herbivore attack. Although total pollinator visitation did not vary with AMF treatment, pollinators exhibited taxon-specific responses, with honey bees, bumble bees, and Lepidoptera all responding differently to AMF treatments. Flower number and size were unaffected by treatments, suggesting that differences in pollinator preference were driven by other floral traits. Mycorrhizae influenced leaf K and Na, but these differences in leaf nutrients did not correspond to variation in herbivore attack. Overall, we found that AMF indirectly influence both antagonistic and mutualistic insects, but impacts depend on the identity of both the fungal partner and the interacting insect, underscoring the context-dependency of plant–AMF interactions.

## INTRODUCTION

Plants interact with a variety of organisms both above and below the soil surface. Belowground interactions between plants and other organisms influence, and are influenced by, interactions aboveground (Bardgett and Wardle, 2003; Wardle et al., 2004; van der Putten et al., 2009). Among the most abundant and widespread soil microbes are arbuscular mycorrhizal fungi (AMF), members of the phylum Glomeromycota that form associations with plant roots and exchange nutrients, such as phosphorus and nitrogen, for plant-derived carbon (Smith and Read, 2008). These globally important fungi interact with 60–80% of terrestrial plant species (Smith and Smith, 2011) and generally confer growth and fitness benefits (Smith and Read, 2008). The impacts of AMF on host plants are usually evaluated based on these direct effects alone. However, the direct effects of AMF on plants may also alter plant traits that mediate interactions between plants and insects, such as pollinators or herbivores, with important consequences for plant fitness (Wolfe et al., 2005; Koricheva et al., 2009; Vannette and Rasmann, 2012).

Colonization by AMF can affect floral traits such as flower number (Schenck and Smith, 1982; Lu and Koide, 1994; Gange et al., 2005) and size (Gange and Smith, 2005; Kiers et al., 2010; Varga and Kytöviita, 2010), as well as nectar and floral volatile characteristics (Gange et al., 2005; Kiers et al., 2010; Becklin et al., 2011). Although the number of studies measuring AMF effects on pollinator visitation to plants in the field is very limited, they have demonstrated that AMF can influence pollinator behavior. Wolfe et al. (2005) and Gange and Smith (2005) both found increased visitation to plants inoculated with AMF compared to non-mycorrhizal plants, and the latter study found that this pattern was consistent among both hymenopteran and dipteran pollinators. In another experiment, the preferences of these two taxonomic groups differed depending on the AMF species used to inoculate the plant (Varga and Kytöviita, 2010). Thus, the direction and magnitude of AMF impacts on plant–pollinator interactions likely depend on both the pollinator and the AMF species colonizing the plant (Gehring and Bennett, 2009).

Insect herbivory is also frequently influenced by AMF colonization (Koricheva et al., 2009), and these effects may be due to mycorrhizal effects on plant biomass, nutrient content, or defenses (Bennett et al., 2006). For example, increased nutrient content of mycorrhizal plants may increase their quality as a food source for herbivores, but the resources made available by this interaction may also be allocated toward defense against herbivores (Vannette and Hunter, 2011). Additionally, AMF may also play an important role in induced resistance of plants against insects by priming the jasmonic acid-dependent responses of plants to herbivory (Pozo and Azcón-Aguilar, 2007; Koricheva et al., 2009; Jung et al., 2012). Regardless of the underlying mechanisms, AMF can indirectly affect plant fitness through changes in herbivory.

The effects of AMF on pollination or herbivory are likely to differ among AMF species or strains. For example, both constitutive and induced levels of defensive chemicals in *Plantago lanceolata* varied among plants inoculated with three different AMF species (Bennett et al., 2009). In a recent study, performance of herbivores feeding on *Fragaria vesca* varied when plants were inoculated with different strains of the AMF *Rhizophagus irregularis *(Roger et al., 2013). These results underscore the importance of examining multiple species in AMF–plant–insect interactions to understand the variation in indirect mycorrhizal effects (Gehring and Bennett, 2009). There are additional challenges to studying insect responses to mycorrhizal variation in a realistic field setting. For example, most field studies of these interactions have manipulated AMF by applying fungicide to plots and observing insect responses (Koricheva et al., 2009). Although this is an effective method of eliminating AMF from experimental plots, there may be unintended effects by altering non-mycorrhizal fungi and other soil organisms.

In this study we tested the hypothesis that plant–AMF interactions belowground influence aboveground interactions between plants, herbivores and pollinators. We used an inoculation experiment to manipulate multiple species/strains of AMF in the roots of *Cucumis sativus* (cucumber, Cucurbitaceae). We transferred plants to an agricultural field setting, measured traits that may affect plant reproduction directly and indirectly, and determined pollinator and herbivore preferences. Although we made no specific predictions about the impacts of each inoculum on plant–insect interactions, we hypothesized that AMF-free plants would have reduced pollinator visitation and based on previous similar studies (Gange and Smith, 2005; Wolfe et al., 2005). Given the role of AMF in induced defenses of *C. sativus *(Barber, 2013), we also expected greater herbivory on these non-mycorrhizal plants.

## MATERIALS AND METHODS

### STUDY SYSTEM

*Cucumis sativus* is a widely cultivated, monoecious annual vining plant that associates with multiple species of AMF. Mycorrhizae can influence flowering, fruit production, photosynthesis rates, and disease resistance in *C. sativus* (Trimble and Knowles, 1995; Valentine et al., 2001; Hao et al., 2005; Kiers et al., 2010). Flowers of *C. sativus* open for a single day and are pollinated by a variety of generalist pollinators, including honey bees (*Apis mellifera*, Apidae), bumble bees (*Bombus* spp., Apidae), solitary bees (e.g., Halictidae), butterflies, and hoverflies (Syrphidae; Barber et al., 2012). All of these pollinators are common in western Massachusetts, USA, where this study took place. Throughout much of eastern North America, the most important herbivore of *C. sativus* is the specialist *Acalymma vittatum* (Chrysomelidae), which accounts for virtually all leaf damage at the study site (Barber et al., 2012). *Acalymma vittatum* larvae feed on root tissue of host plants prior to pupation.

### EXPERIMENTAL DESIGN

We surface sterilized *C. sativus* seeds (Marketmore 76, Johnny’s Selected Seeds, Winslow, ME, USA) using 5% bleach solution and germinated them in steam-sterilized potting mix (Fafard organic mix, Agawam, MA, USA). At transplanting, we inoculated 192 seedlings with one of six AMF treatments. For our fungal treatments, we chose three closely related fungal species: *Glomus clarum, G. custos *(strain 010 Mycovitro), and *R. irregularis *[strain 09 Mycovitro, see Stockinger et al. (2009) for discussion of *G. intraradices* reclassification] in the Glomeraceae. These three species were chosen because they have been shown to differ in the growth benefits (i.e., P and N benefits) they confer to various host plants (Kiers et al., 2011; Ortas and Akpinar, 2011; Verbruggen et al., 2012; Hart et al., 2013). The use of closely related AMF allowed us to focus on fungal cooperative strategy while excluding differences associated with radically contrasting life-history traits (Denison and Kiers, 2011). These fungal species were applied in liquid form (1 mL inoculum applied directly to seedling roots) from solubilized *in vitro* root cultures obtained from Estacion Experimental del Zaidin, Consejo Superior de Investigaciones Cientificas, Granada, Spain. Fungal species were applied singly, but we also included a mixed treatment, which was composed of equal volumes (333 μL) of these three species together. The inoculum of these three species did not contain significantly different densities of spores (100 μL samples, *n* = 5 each, mean ± SE, *G. clarum* 11.8 ± 3.1; *G. custos *10.6 ± 1.6; *R. irregularis* 8.2 ± 2.4, *F*_2,12_ = 0.55, *P* = 0.589).

While these strains are well-characterized laboratory strains, we were also interested in studying the effects of commercial inoculum that farmers would apply in the field. Therefore, we included a commercial inoculum also composed of a *R. irregularis* strain (isolate **DAOM** 197198; hereafter we refer to this inoculum as “commercial” to distinguish it from the *R. irregularis* strain 09) produced by Myke Premier Tech Biotechnologies (Rivière-du-Loup, QC, Canada). The commercial inoculum was on a perlite mixture that was added into the transplant pot (60 mL, ca. 120 spores). We also included a non-mycorrhizal control, in which plants received 1 mL water. The result was six AMF treatments (three single species, one mixture, one commercial inoculum, and one control). Both liquid and perlite inocula also contained colonized root fragments and mycorrhizal hyphae as well as spores. Although perlite from the commercial treatment represented only about 2.4% of the total volume of soil in the pot, it could potentially influence plants through changes in soil structure and increased drainage. However, perlite does not influence soil cation exchange capacity and has little effect on soil nutrients.

To create a common soil growing medium for all plants, we mixed soil from an agricultural field at the study site with an equal volume of sand and autoclaved the mixture to sterilize it; characteristics of this soil mixture are presented in **Table 1**. We filled 2.5 L bleach-sterilized pots, lined with bleach-sterilized plastic mesh, with the sterile soil mixture and transplanted inoculated seedlings on 1–2 June 2011. We transferred half of the plants to an agricultural field at the UMass Agricultural Research Center (South Deerfield, MA, USA) on 6 June to determine how AMF treatments affected leaf nutrient content in a field setting. We arranged plants in 16 blocks (rows) of six plants each (one plant/treatment/block, all plants spaced 2.5 m apart). We placed each pot into a black plastic tray filled with sand to create a barrier between the pot and AMF in the field soil. We allowed plants to grow under field conditions for 22 days, after which leaf tissues were collected and dried for nutrient analysis. We watered plants daily unless there was rainfall in the past 24 h.

**Table 1:** Characteristics of sterilized soil mixture (mean ± 1 SE, n = 2).

Soil characteristic	
pH	7.15 ± 0.15
nitrate	3.0 ± 0.0
% organic matter	0.9 ± 0.1
CEC	3.65 ± 0.55
P	22.5 ± 6.5
K	69.0 ± 0.0
Ca	918 ± 134
Mg	38.5 ± 0.5
Al	19.0 ± 1.0
B	0.1 ± 0.0
Mn	21.8 ± 5.8
Zn	1.2 ± 0.7
Cu	0.9 ± 0.0
Fe	2.0 ± 0.1
S	34.0 ± 10.8
Pb	2.95 ± 0.5

*CEC = cation exchange capacity; all measurements (other than pH, % organic matter, and CEC) are ppm.*

The remaining 96 plants were maintained in a greenhouse with natural light and locations rotated on greenhouse benches weekly. On 1 July we transported these plants to the same field and arranged the blocks in 16 rows. Although it is possible fungal spores could enter pots of both groups through the air, we expect these effects to be minimal given the short duration plants were in the field (22–25 days). Keeping these plants in the greenhouse prior to transferring them to the field prevented early season herbivory, which affects interactions with AMF, herbivores, and pollinators (Barber et al., 2011, 2012). This ensured that responses were due to AMF treatments and not interactions between AMF and early season herbivores. All responses other than leaf nutrient content were measured in this second group of plants.

### PLANT MEASUREMENTS

Dried leaf tissue from the first set of plants was ground and analyzed by the UMass Soil and Plant Tissue Testing Laboratory to determine nutrient content (leaf N, P, K, and Na). On the second set of plants, we counted flowers daily 5 days/week and measured flower petal length and width on the first two male flowers produced by each plant. A single plant produced its first flower in the greenhouse, prior to transportation, but we measured all other flowers in the field. On 25–26 July, we harvested all plants in the second set, separating root and shoot tissues. We dried plants at 60°C and measured root and shoot mass of each plant. We froze a small sample of roots collected prior to drying from each plant in four blocks. We stained these frozen root samples with trypan blue and quantified AMF colonization using the magnified gridline intersect method (McGonigle et al., 1990).

### POLLINATION

We surveyed pollinator visitation to plants on 14 days from 5 to 22 July for a total of 63.25 person-hours of observation. We performed all surveys between 0930 and 1400, when pollinators were most active at the site. We followed individual pollinators within the experimental plot and used handheld digital voice recorders to record pollinator taxon and plants visited, number of flowers probed per visit, and time spent per flower in seconds. Individual pollinators were followed as long as possible or until they left the plot. We calculated the proportion of flowers probed per visit as the observed flower probes divided by the total number of open flowers. We analyzed number of visits and proportion of flowers probed for all pollinators combined and for the five most common pollinator taxa independently: honey bees (*Apis mellifera*), bumble bees (*Bombus* spp.), small bees (family Halictidae), Lepidoptera, and hoverflies (family Syrphidae).

### HERBIVORY

We surveyed herbivory on 5 and 12 July. On each plant, we used clear plastic grids to measure the area consumed on the three most recent fully expanded leaves. Because total leaf damage was low during the surveys (three quarters of the plants had <5% herbivory in the first survey, and nearly all in the second survey), leaves were categorized as damaged and undamaged and analyzed using a binomial model (see Analysis).

### ANALYSIS

We used generalized linear mixed models (GLMM) to determine if AMF inoculum affected plant characteristics and interactions with herbivores and pollinators. We fit random intercepts models using lmer() in the lme4 package for Poisson and binomial models (Bates et al., 2012) and lme() in the nlme package for Gaussian models (Pinheiro et al., 2010) of R 2.15.0 (R Development Core Team, 2012), treating block as a random effect and AMF treatment as a fixed effect. For count data (flower number, pollinator visits, and pollinator probes) we used Poisson errors and log link function and individual-level random effects to account for overdispersion (Agresti, 2002). We analyzed both herbivore survey dates together by including date as a fixed factor and plant as a random factor (in addition to block). Binomial data were presence/absence of damage on each of the three leaves examined on each date. For each survey date, there were four possible responses (0, 1, 2, or 3 out of 3 leaves damaged). This is equivalent to a repeated measures analysis, but in a binomial linear model framework. For continuous response variables (flower size, aboveground and belowground plant growth, proportion flowers probed), we used Gaussian errors and identity link.

The goal of this experiment was to determine whether AMF inoculum influenced plant traits and plant–insect interactions; we did not have specific predictions about how individual inocula might differ compared to each other. We used likelihood ratio tests to compare models with and without AMF treatment, which compare likelihood ratios to a χ^2^ distribution. When this test was significant at *P* < 0.05, we compared each single species inoculum (*G. clarum, G. custos, R. irregularis, *and commercial) to the AMF-free control. We also tested one additional *a priori *hypothesis contrasting the mixture treatment of *G. clarum, G. custos, *and *R. irregularis *with the three independent treatments of these species combined. This tests if these AMF species have additive or interactive effects when combined; a significant contrast indicates that the species in combination interact to produce a response different from average responses to single species colonizations. We used the multcomp package (Hothorn et al., 2008) to perform contrasts, adjusting *P*-values for multiple comparisons using the Westfall method, a resampling procedure that can be applied to Gaussian, binomial, and Poisson models (Westfall, 1997), as implemented in the glht() function of multcomp.

## RESULTS

### PLANT MEASUREMENTS

**FIGURE 1:**
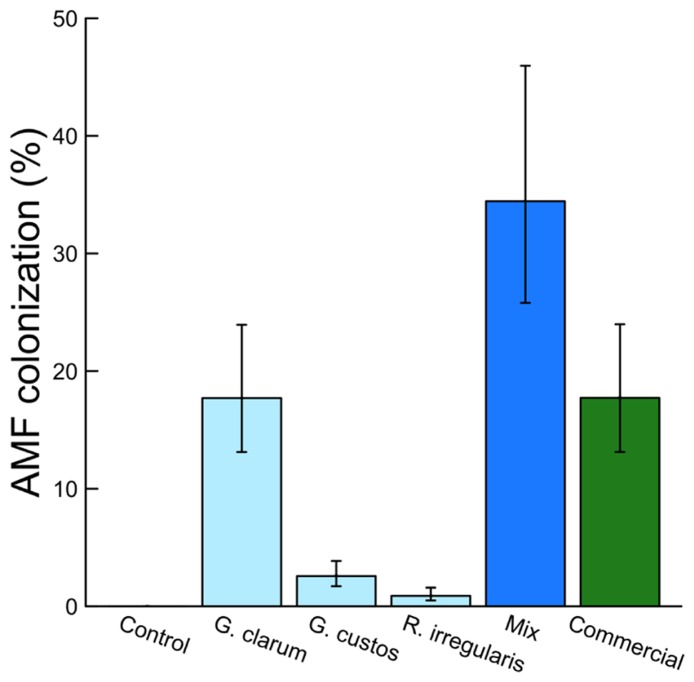
**Effects of AMF inoculation treatments on percent AMF colonization. Values are fitted means ± 1 SE**.

**FIGURE 2:**
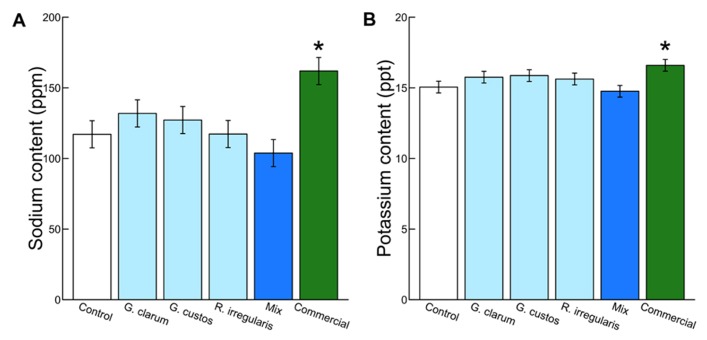
**Effects of AMF inoculation treatments on (A) leaf Na content and **(B)** leaf K content.** Values are fitted means expressed in parts per million ± 1 SE for Na and parts per thousand ± 1 SE for K. Asterisk indicates significant difference from non-mycorrhizal control.

Colonization varied significantly among the inoculation treatments (χ^2^ = 46.51, df = 5, *P* < 0.001), with the mix inoculum treatment showing the highest colonization levels. No AMF structures were observed in control plants (**Figure 1**). AMF inoculation treatment did not affect total plant biomass (χ^2^ = 7.59, df = 5, *P* = 0.181) or root:shoot ratio (χ^2^ = 4.46, df = 5, *P* = 0.486). There were also no differences in total flower number (χ^2^ = 4.40, df = 5, *P* = 0.492) or flower petal size (χ^2^ = 3.34, df = 5, *P* = 0.634). AMF treatments did affect leaf nutrient content, with a significant effect on leaf K and Na and a marginally significant effect on leaf P (**Table 2**). Leaf K and Na were significantly increased by commercial AMF compared to control (**Table 2**, **Figure 2**); no other treatments were significantly different from the control.

**Table 2:** Results of GLMM analyses of AMF treatment effects on leaf nutrient content.

	χ^**2**^/Wald *Z*	*P*
Leaf N	2.49	0.778
Leaf P	9.56	0.089
Leaf K	**12.97**	**0.024**
Control vs. *G. clarum*	1.32	0.313
Control vs. *G. custos*	1.55	0.283
Control vs. *R. irregularis*	1.08	0.313
Control vs. commercial	**2.92**	**0.017**
Mixture vs. single	2.30	0.076
Leaf Na	**19.35**	**0.002**
Control vs. *G. clarum*	1.16	0.514
Control vs. *G. custos*	0.79	0.643
Control vs. *R. irregularis*	0.02	0.986
Control vs. commercial	**3.51**	**0.002**
Mixture vs. single	2.08	0.127

*Tests of overall effect of AMF treatment are χ^**2**^ tests; when significant, contrasts used Wald *z* tests with *P*-values adjusted for multiple comparisons using the Westfall method (see Analysis).*

*Notes: d.f. = 5 for χ^2^ test, d.f. = 1 for all Wald *z* tests.*

*Bold indicates results with P <0.05.*

### POLLINATION

**FIGURE 3:**
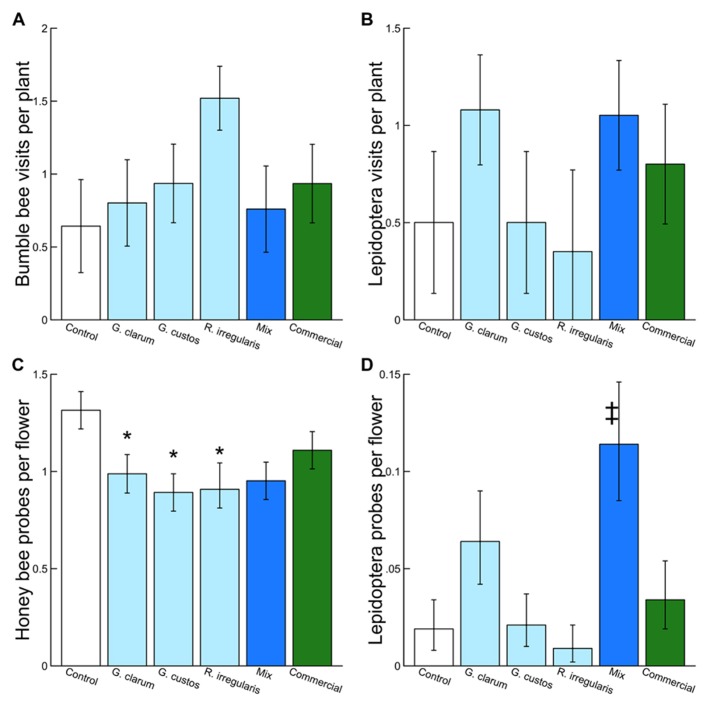
**Effects of AMF inoculation treatments on (A) bumble bee visits per plant, (B) Lepidoptera visits per plant, (C) honey bee probes per flower, and (D) Lepidoptera probes per flower.** Values are fitted means ± 1 SE. Asterisks indicates significant differences based on *a priori *contrasts between non-mycorrhizal control and each single species inoculum (*G. clarum, G. custos, R. irregularis, *and commercial). Double dagger indicates significant difference based on *a priori *contrast between the mixed inoculum and its component single-species inocula.

We observed 2,498 plant visits by pollinators and 4,254 individual flower probes. Although AMF treatments did not influence total pollinator visitation, visitation of several pollinator taxa varied with AMF inoculum (**Table 3**). There was a significant effect of AMF treatment on visitation by bumble bees (**Figure 3A**) and Lepidoptera (**Figure 3B**) and a marginally significant effect on honey bee visitation. Bumble bee visitation was greatest to plants inoculated with *R. irregularis*; this difference was marginally significant after controlling for multiple comparisons. Similarly, although AMF affected Lepidoptera visits overall, visits to particular treatments did not differ in any individual contrasts (**Table 3**).

**Table 3:** Results of GLMM analyses of AMF treatment effects on total number of pollinator visits per plant and proportion of flowers probed, by pollinator taxa.

	Total pollinator visits		Proportion flowers probed	
	χ^**2**^/Wald *Z*	*P*	χ^**2**^/Wald *Z*	*P*
All pollinators	2.00	0.850	6.57	0.255
Honey bees (*Apis mellifera*)	9.70	0.084	**14.37**	**0.013**
Control vs. *G. clarum*	–	–	**2.51**	**0.035**
Control vs. *G. custos*	–	–	**3.31**	**0.004**
Control vs. *R. irregularis*	–	–	**3.18**	**0.006**
Control vs. commercial	–	–	1.62	0.201
Mixture vs. single	–	–	0.22	0.828
Bumble bees (*Bombus* spp.)	**11.20**	**0.048**	1.18	0.947
Control vs. *G. clarum*	0.53	0.720	–	–
Control vs. *G. custos*	0.94	0.720	–	–
Control vs. *R. irregularis*	2.36	0.075	–	–
Control vs. commercial	0.94	0.720	–	–
Mixture vs. single	1.01	0.720	–	–
Lepidoptera	**13.52**	**0.019**	**24.17**	**<0.001**
Control vs. *G. clarum*	1.98	0.144	2.14	0.105
Control vs. *G. custos*	0.00	1.000	0.13	0.900
Control vs. *R. irregularis*	0.72	0.701	0.79	0.706
Control vs. commercial	1.15	0.515	0.88	0.706
Mixture vs. single	2.12	0.135	**4.01**	**<0.001**
Halictidae	5.79	0.327	9.13	0.104
Syrphidae	2.45	0.784	2.93	0.711

*Tests of overall effect of AMF treatment are χ^**2**^ tests; when significant, contrasts used Wald *z* tests with *P*-values adjusted for multiple comparisons using the Westfall method (see Analysis). *Notes: *d.f. = 5 for all χ^2^ tests, d.f. = 1 for all Wald *z* tests. *

*Bold indicates results with P <0.05.*

Arbuscular mycorrhizal fungi treatments also did not affect the proportion of flowers probed per plant by pollinators in total but did affect the proportion probed by honey bees and by Lepidoptera (**Table 3**). Honey bees probed a significantly lower proportion of flowers on plants inoculated with each of the single species inocula compared to AMF-free control plants, and honey bee probes were no more likely on mixture plants than single species plants (**Figure 3C**). Lepidoptera tended to probe a higher proportion of flowers on plants inoculated with *G. clarum* or the three species mixture, although only the mixture vs. single species contrast was significant (**Figure 3D**).

### HERBIVORY

**FIGURE 4:**
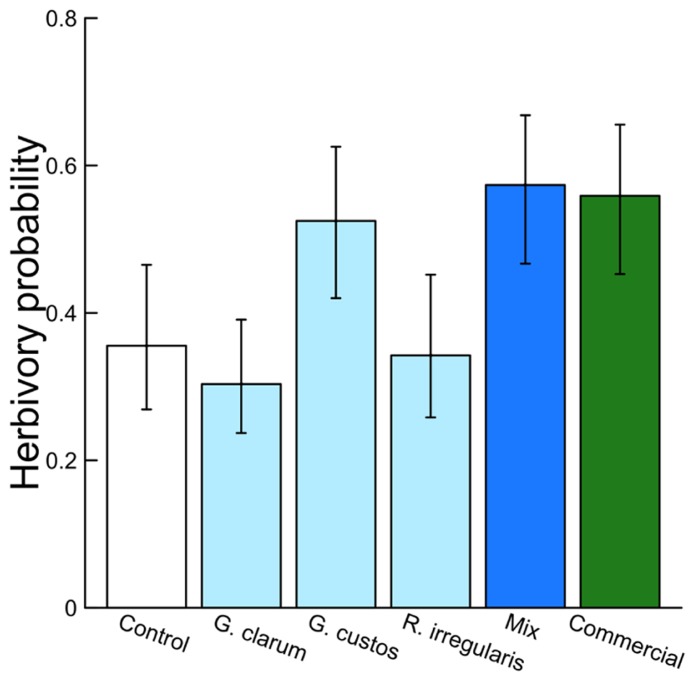
**Effects of AMF inoculation treatments on probability of herbivore attack.** Values are fitted means ± 1 SE, transformed from logits to probability for ease of interpretation.

Arbuscular mycorrhizal fungi significantly affected herbivore damage (**Table 4, Figure 4**), a result that was likely driven by higher probability of damage to mixed inoculum and commercial inoculum plants. Date was also significant (χ^2^ = 96.00, *P* < 0.001), because herbivore damage was much more prevalent on the earlier survey date. However, after adjusting for multiple comparisons, no AMF treatment contrasts were significant.

**Table 4:** Results of GLMM analyses of AMF treatment effects on herbivory.

	χ^**2**^/Wald *Z*	*P*
Herbivory	**12.49**	**0.029**
Control vs. *G. clarum*	0.53	0.820
Control vs. *G. custos*	1.62	0.247
Control vs. *R. irregularis*	0.13	0.896
Control vs. commercial	1.95	0.158
Mixture vs. single	2.23	0.107

**Notes: *d.f. = 5 for χ^2^ test, d.f. = 1 for all Wald *z* tests.*

*Bold indicates results with P <0.05.*

## DISCUSSION

Species interactions can be very context-dependent, and outcomes will vary depending on various biotic and abiotic factors. This is evident in natural systems as well as in agroecosystems (Tscharntke et al., 2008). In agricultural fields, mycorrhizal and other symbioses may modify a range of plant traits that alter the nature or frequency of plant–insect interactions important for plant reproduction. This can happen via direct effects on plant growth, nutrition, and other traits. Here, we found that associations between plants and AMF influenced aboveground plant interactions with both pollinators and herbivores and that these effects differed among both AMF and insect species, highlighting the context-dependent nature of these interactions.

Colonization varied significantly among AMF treatments, with the highest colonization (fungal structures present in >34% of root length on average) by the mix treatment containing three AMF species. This level is greater than the average colonization by any of the component species alone and greater than the sum of these single species, suggesting there may be synergistic interactions that benefit the fungi when multiple species are present. The lowest colonization, other than the AMF-free control, was by *R. irregularis. *This was much lower than the commercial inoculum, also a strain of *R. irregularis, *illustrating the wide variation in colonization potential possible among even taxa categorized as conspecific (Stockinger et al., 2009; Roger et al., 2013).

### POLLINATION

Inoculation by different AMF species influenced the behavior of several taxonomic groups of insect pollinators. Honey bees, bumble bees, and Lepidoptera behavior varied with inoculation treatment, and the patterns of visitation and flower probing differed among these groups. Differences in visitation to plants by bumble bees was driven by apparent greater preference for plants inoculated with *R. irregularis, *although this contrast was marginally significant after adjustment. Similarly, there was a trend toward greater preference by Lepidoptera for plants inoculated with *G. clarum*. While the decision to begin foraging on a plant (i.e., a plant visit) may be determined by long- and short-range cues, the proportion of flowers probed may be a better indicator of pollinator assessment of floral quality (Mitchell and Waser, 1992). Pollinators are expected to probe a greater number of flowers on a high-reward plant. Conversely, a visit to a less-rewarding plant may be terminated before all flowers have been visited.

We found that the proportion of flowers probed by both honey bees and Lepidoptera varied with AMF inoculation treatment. Honey bee flower probing rates were significantly reduced on plants that had been inoculated with single species of AMF compared to non-mycorrhizal controls; this is surprising given that AMF usually increase floral reward and pollinator preference (Gange and Smith, 2005; Gange et al., 2005; Aguilar-Chama and Guevara, 2012). Lepidoptera flower probes were significantly greater for mixture plants than single species inocula plants combined, suggesting that the three AMF had interactive effects on floral traits that increased Lepidoptera preference.

Previous studies have found that the effects of AMF on pollinator behavior differ among plant species and pollinator taxa. Gange and Smith (2005) compared pollinator visitation to mycorrhizal and non-mycorrhizal individuals of three plant species. Although AMF generally increased visitation the effect differed with specific combinations of pollinator taxa and plant species, with increased Hymenoptera visitation to two species and Diptera visitation to the other species. Similarly, we show that visitation or flower probes varied by taxa (bumble bees, honey bees, and Lepidoptera) and inoculum type. This suggests that AMF species or strains alter plant traits in different ways, and that these pollinator taxa differ in their response to these traits. In a study that manipulated two AMF species, including *R. irregularis*, pollinator visitation increased with both AMF, although only a subset of the pollinator community was examined (Wolfe et al., 2005). Of the few prior experiments on AMF effects on pollinator behavior, only one (Varga and Kytöviita, 2010) both manipulated AMF species identity and examined multiple pollinator taxa, as we did here. Interestingly, they found reduced Syrphidae visitation to female *Geranium sylvaticum* (Geraniaceae) when plants were inoculated with one species of AMF, compared to control and the other AMF species. They also showed reduced visitation by small Hymenoptera to plants inoculated with the other species. Syrphidae and small Hymenoptera (here, Halictidae) were also common visitors in our experiment, but we found no effects of AMF inocula on visitation or flowers probed. Taken together, these results indicate that different AMF species likely have distinct effects on floral traits and that pollinators have taxa-specific responses to these trait changes.

Pollinators responded to AMF treatments, despite the lack of treatment effects on the floral traits we measured. Gange and Smith (2005) attributed increased pollinator visitation on mycorrhizal plants to greater inflorescence number or size for two Asteraceae species. However, our AMF treatments did not affect flower number or size, which contrasts with many studies that find increased flower production in plants associating with AMF (Bryla and Koide, 1990; Perner et al., 2007; Varga and Kytöviita, 2010). In previous work *R. irregularis* increased male flower production and flower diameter in *C. sativus, *although the effect on flower size was eliminated by addition of methyl jasmonate (Kiers et al., 2010). Herbivore attack triggers jasmonic acid responses in plants (Farmer et al., 2003), so insect herbivory on our plants in the field may have erased any positive effects of AMF on floral traits. However, herbivore attack was not significantly correlated with total flower production or male flower size (data not shown). Nectar production and composition and floral volatiles can also have profound effects on pollinator behavior (Schemske and Bradshaw, 1999; Dudareva and Pichersky, 2006; Adler, 2007), but were not measured in this study. Effects of AMF on nectar quantity and quality vary among plant species (Gange and Smith, 2005; Becklin et al., 2011), and AMF use of plant photosynthates may reduce plant carbohydrate availability for nectar (Laird and Addicott, 2007). Nectar production in *C. sativus, *like flower size, was also reduced by methyl jasmonate application (Kiers et al., 2010), so herbivory may interact with mycorrhizal status to affect nectar. Floral scent from volatile production affects pollinator attraction, and experimental elimination of soil fungal communities altered volatile production in *Polemonium viscosum *(Becklin et al., 2011). However, inoculation with commercial AMF or farm AMF communities had no effect on *C. sativus* volatiles compared with non-mycorrhizal controls (Barber et al. unpublished data), suggesting that this trait may not explain the indirect effects of AMF on pollinators observed here.

### HERBIVORY

Mycorrhizal treatment significantly affected the probability of herbivore damage to leaves, with probability of attack varying from 0.3 in plants inoculated with *G. clarum* to nearly 0.6 in plants with a mixture of AMF species. Control plant herbivory was intermediate, so individual treatments did not differ significantly from control (**Figure 4**). Inoculation affected leaf nutrient content, but surprisingly not P or N, the nutrients that are most frequently studied in plant–AMF research. Rather, commercial AMF inoculum significantly increased leaf K and Na content relative to non-mycorrhizal plants, although the increase in K was modest. Recent work has emphasized the potential importance of less-studied elements that exist in both organic molecules and ionic forms, but are essential to herbivores (Behmer and Joern, 2012; Joern et al., 2012). Sodium can be limiting for insect herbivores because it occurs in low concentration in plant tissues (Kaspari et al., 2008; Behmer and Joern, 2012; Chavarria Pizarro et al., 2012), and potassium was also identified as a predictor of insect herbivore abundance (Joern et al., 2012). If AMF alter plant concentrations of these elements (in organic or inorganic forms) that are important to insect nutrition, it may provide an additional mechanism of indirect mycorrhizal effects on insect herbivore preference and performance. Future work could address whether the magnitudes of these differences in elemental content (20–50 ppm Na, 1–2 ppt K) are sufficient to influence insect herbivore preference or performance.

Treatment effects on herbivory could also be caused by AMF influences on plant defenses. Colonization of plant roots by AMF is thought to induce both local and systemic responses that allow the plant to respond more rapidly or efficiently to attack by herbivores or pathogens (Jung et al., 2012). Mycorrhization increased induced defenses against a generalist herbivore (*Spodoptera exigua*) in *C. sativus*, with herbivores consuming more leaf tissue on induced mycorrhizal plants without increasing their biomass (Barber, 2013). Given this finding, we would expect reduced herbivory on inoculated plants compared to control plants in this experiment, but instead we found lower herbivory on control plants (**Figure 4**). This may in part be explained by the dominant wild herbivore in this agroecosystem, *Acalymma vittatum* (striped cucumber beetle), a specialist that responds positively to cucurbitacins, the primary defensive chemicals in *Cucumis* and its relatives (Metcalf et al., 1980). The role of AMF in inducing plant defenses may be more important for generalist herbivores than specialists. This hypothesis is supported by a meta-analysis of AMF–herbivore experiments that found more negative effects of mycorrhizae on generalist chewing herbivores than on specialists (Koricheva et al., 2009).

## CONCLUSION

The outcomes of plant–AMF interactions have historically focused on the direct effects of the fungi on plants, such as plant growth or nutrient content. However, plant growth and fitness are also influenced by community members, whose interactions may be modified by AMF-driven changes in plant traits. Here we show that colonization by different AMF species has consequences for pollinator visitation and herbivory in an agroecosystem, but these effects vary with both AMF and insect identity. For AMF–plant–pollinator interactions, future work should focus on the multiple floral traits that can mediate pollination, including how different AMF species (or AMF communities from different ecological contexts) influence floral traits like visual cues, nectar production and composition, and floral scent. A more detailed understanding of AMF effects on these flower traits will allow better predictions of pollinator responses based on the floral signals used by different pollinator taxa. Similarly, understanding AMF effects on herbivory will require experiments that measure plant nutrients and chemical defenses in a field setting or controlled, but ecologically realistic, laboratory conditions. Our results demonstrate the potential for above- and belowground communities to interact in complex ways via species-specific responses of insects and their effects on plant fitness. Thus, even in relatively simple agroecosystems, diversity may provide an important buffer maintaining critical species interactions.

## Conflict of Interest Statement

The authors declare that the research was conducted in the absence of any commercial or financial relationships that could be construed as a potential conflict of interest.
